# Towards Void Hole Alleviation by Exploiting the Energy Efficient Path and by Providing the Interference-Free Proactive Routing Protocols in IoT Enabled Underwater WSNs

**DOI:** 10.3390/s19061313

**Published:** 2019-03-15

**Authors:** Muhammad Awais, Nadeem Javaid, Amjad Rehman, Umar Qasim, Musaed Alhussein, Khursheed Aurangzeb

**Affiliations:** 1 Department of Computer Science, COMSATS University Islamabad, Islamabad 44000, Pakistan; amawais@hotmail.com; 2 College of Computer and Information Systems, Al Yamamah University, Riyadh 11512, Saudi Arabia; drrehman70@gmail.com; 3 Cameron Library, University of Alberta, Edmonton, AB T6G 2J8, Canada; umar.qasim@ualberta.ca; 4 Computer Engineering Department, College of Computer and Information Sciences, King Saud University, Riyadh 11543, Saudi Arabia; musaed@ksu.edu.sa (M.A.); kaurangzeb@ksu.edu.sa (K.A.)

**Keywords:** energy efficient, void hole, shortest path based routing, EC, depth

## Abstract

Nowadays, the Internet of Things enabled Underwater Wireless Sensor Network (IoT-UWSN) is suffering from serious performance restrictions, i.e., high End to End (E2E) delay, low energy efficiency, low data reliability, etc. The necessity of efficient, reliable, collision and interference-free communication has become a challenging task for the researchers. However, the minimum Energy Consumption (EC) and low E2E delay increase the performance of the IoT-UWSN. Therefore, in the current work, two proactive routing protocols are presented, namely: Bellman–Ford Shortest Path-based Routing (BF-SPR-Three) and Energy-efficient Path-based Void hole and Interference-free Routing (EP-VIR-Three). Then we formalized the aforementioned problems to accomplish the reliable data transmission in Underwater Wireless Sensor Network (UWSN). The main objectives of this paper include minimum EC, interference-free transmission, void hole avoidance and high Packet Delivery Ratio (PDR). Furthermore, the algorithms for the proposed routing protocols are presented. Feasible regions using linear programming are also computed for optimal EC and to enhance the network lifespan. Comparative analysis is also performed with state-of-the-art proactive routing protocols. In the end, extensive simulations have been performed to authenticate the performance of the proposed routing protocols. Results and discussion disclose that the proposed routing protocols outperformed the counterparts significantly.

## 1. Introduction

Nowadays, the Internet of Things Enabled Underwater Wireless Sensor Network (IoT-UWSN) is an emerging communication framework. The aforementioned paradigm allows a varied range of distinctive applications, i.e., scientific ocean sampling, disaster prevention, inhabitant monitoring, assisted navigation, etc. [1,2]. IoT-UWSN is different from the terrestrial network in many aspects. For example:

1. Localization in IoT-UWSN is quite difficult from terrestrial networks.
2. The dynamically changing environment is difficult to handle in IoT-UWSN as compared to terrestrial sensor networks.
3. IoT-UWSN uses acoustic signals for data transmission. On the contrary, radio signals are used in the terrestrial environment for packets transmission.
4. In IoT-UWSNs, sensor nodes are deployed sparse relative to the terrestrial environment.
5. In IoT-UWSNs, the sensor nodes have limited energy. Therefore, after the sensors deployment in an underwater environment, it is difficult to replace the node’s battery as compared to terrestrial ones [3].

The characteristics of limited energy, high End to End (E2E) delay, high bit error rate and limited available bandwidth are the fundamental challenges in the underwater environment. In addition, the use of acoustic signals in the water depth make these issues more complicated, especially energy efficiency [4].

Radio and optical waves are not feasible in UWSNs because they get absorbed and scatter in the water rapidly. On the contrary, acoustic signals are preferred in the underwater environment because of their low absorption and scattering rate. However, they face a high E2E delay due to low propagation speed. Acoustic signals are strong near the source and become weak as moving away from the source [4]. These signals are impaired with noises in the sea due to which signals get attenuated during propagation. This attenuation causes a high bit error rate and if this error is more than a certain threshold, ultimately the packet gets dropped. Furthermore, the major noises in the sea come from the shipping, wind and turbulence. However, intuitively at the bottom of the sea, the environment is much quieter as compared to the sea surface.

Due to these reasons, different protocols have been proposed by the researchers in IoT-UWSNs, i.e., Depth Base Routing (DBR) protocol is proposed to avoid the void hole issue in UWSN [5]. In the same way, Weighting Depth Forwarding Area Division DBR (WDFAD-DBR) protocol is suggested to handle the void hole problem [6]. However, it considers only 2-hop neighbor information for forwarder node selection to minimize the chance of void hole presence. Moreover, some backward transmission based protocols are also proposed to tackle the issues of the void hole occurrence, interference and collision-free transmission [7]. However, these strategies have not outperformed. Therefore, the above analysis shows that there is a strong need to enhance these routing protocols in terms of high Packet Delivery Ratio (PDR), low packet loss ratio with minimum Energy Consumption (EC) and minimum E2E delay.

Therefore, in the current work, two proactive routing protocols are implemented. In both protocols, the sender based approach is implemented to minimize the probability of void hole occurrence for reliable data delivery. Therefore, multi-hop communication is adapted for reliable data delivery and the shortest routing path selection for minimum EC to extend the network lifespan. The contributions of this work are:

1. The Energy-efficient Path-based Void hole and Interference-free Routing (EP-VIR-Three) protocol is proposed, which selects the next forwarder node by checking 3-hop neighbors information (the 3-hop forwarder nodes information is based on the depth difference of current and the previous node) to avoid the void hole problem with enhanced PDR.
2. The Bellman-Ford Shortest Path-based Routing (BF-SPR-Three) protocol is proposed, which selects the next forwarder node using ‘Bellman-Ford (BF)’ algorithm. This algorithm provides the shortest and fastest path for routing. In addition, the binary tree approach with horizontal layering concept is implemented for reliable data delivery. Furthermore, it solves the problem of routing loop problem.
3. The problem in this work is tested over multiple simulations and verified by calculating feasible regions for the proposed routing protocols using linear programming. Furthermore, the scalability analysis of the proposed routing protocols is also performed by varying the number of nodes in the UWSN.
4. Comparative analysis is also performed with state-of-the-art proactive routing protocols. Moreover, the proposed protocol shows efficacy in terms of high PDR and minimum EC with affordable E2E delay.

Further, some state-of-the-art routing protocols are reviewed and compared on the basis of some specific parameters, i.e., reliability, packet size and number of hops while communicating in an underwater environment. Moreover, these routing protocols are categorized into three different categories, i.e., energy efficient routing, topology-based routing and void hole avoidance based routing. The achievements, challenges and the limitations of these routing protocols are also discussed below.

### 1.1. Energy Efficiency Based Routing

In this subsection, the energy efficient routing protocols are discussed with their achievements, challenges and limitations. In [8,9,10,11,12,13,14,15,16,17,18,19,20,21], the energy efficient routing protocols are proposed. In the papers [8,10,11,16,17,18,21], authors worked on reliability of Data Packets (DP) from the source node to the destination node. Moreover, for efficient routing, packet size is kept small. Additionally, authors in [10,17] worked on packet size management for reliable and efficient data delivery. The small packet size during data transmission results in fewer collisions during transmission. In addition, mobility management is also an important parameter while designing a routing protocol. This important parameter is focused on by the authors in [8,11,17,19,20,21], respectively. Considering these important parameters, authors in [10,11,16,17,21] performed single hop routing. Meanwhile, multi-hop routing is also considered in [8,9,16,17,18,19,20,21].

The energy effective routing is enhanced by the authors in [8,9,10,11,12,13,14,15,16,17,18,19,20]. In return, the lifetime of the network is improved. PDR is focused in [8,11,17]. In addition, load traffic is balanced by the authors in [10]. Moreover, the protocol proposed in [16] is efficient in handling a large amount of DPs (during dense network). The efficient path selection routing protocol is proposed by the authors in [18]. Aforementioned routing protocols outperformed to achieve their objectives.

However, these routing protocols have some challenges and limitations, i.e., the dense deployment of nodes in [8] and attenuation problem in [9]. Moreover, nodes have to face E2E delay during data delivery. Furthermore, network has to face path loss because of water currents [10,11]. To overcome these issues, additional energy is required which makes the network energy deficient. The authors in [10,11] have not focused on additional EC problem in UWSN, which degrades the network performance. Extra resource allocation and network security are the key challenges in [16,17], respectively. However, the network is facing a high E2E delay. Additionally, both protocols are not performing effectively in the sparse network. Complexity and data loss are the serious challenges in [18,19]. Moreover, extra memory is needed to compute extra calculations in [18,19]. Overhead in routing and security issues with the complex networks are the important challenges in [20,21]. In the end, packet E2E delay and network complexity is increased in the aforementioned papers.

### 1.2. Topology Based Routing

The topology based protocols are elaborated in [9,12,13] with their achievements, challenges and limitations. The protocol implemented in [9] is multi-hop. Moreover, the protocol proposed in [13] is single hop. In both protocols, EC is minimized with enhanced PDR. Moreover, dynamic topology is supposed by the authors in [9], while, in [14], the error resilient network topology is designed for the communication process. However, EC is a bit high due to certain computations.

Topology change is a challenge in [9]. Similarly, the mobility of the sensor nodes is difficult to handle in [12] with a high bit error rate. Minimum attenuation is a serious challenge in [13]. However, E2E delay is increased in the proposed protocol [9]. However, the aforementioned proposed protocols outperform in the predefined underwater environment [12,13].

### 1.3. Void Node Avoidance Based Routing

**Figure 1:**
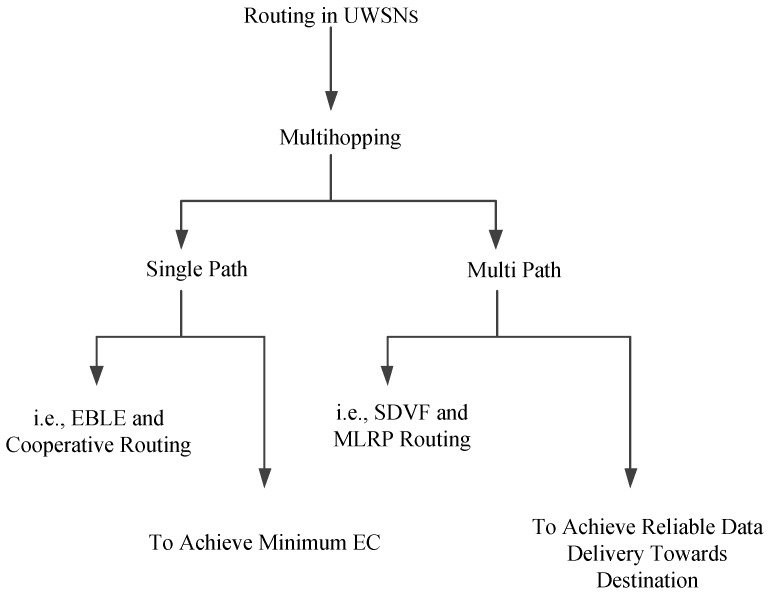
Pictorial description of routing protocols in underwater wireless sensor networks.

The void hole avoidance based protocols are proposed in [22,23,24]. E2E delay is efficiently minimized by the proposed routing protocols. However, the main challenges include low bandwidth and high error rate [22]. Additionally, the computation time of these protocols is bit high [23,24]. The EC in Depth Adjustment (DA) creates an additional void hole which decreases the lifespan of the network [23]. While, in [24], the multiple copies of data are generated, which creates a communication overhead with affordable E2E delay. The pictorial description of the routing protocols in UWSNs is given in Figure 1.

### 1.4. Summarized Literature Review of the Existing Routing Protocols

Nowadays, the main focus of the researchers is on EC and lifetime of the underwater network. The main reasons for EC are the void hole occurrence, interferences and collisions between the DPs. Many of the researchers worked on EC minimization, location error resilience and E2E delay minimization. However, the features of these protocols vary with the change in the requirements in an underwater environment. The routing protocols perform transmission using both multi path and single path. The reason for multi path routing is the reliability in data transmission. In this type of routing, the EC is bit high, which is a key challenge for the researchers in UWSNs. On the contrary, the single path routing is used for minimum EC, which enhances the network lifetime. Table 1 presents the literature review of the baseline protocols.

### 1.5. Reasons for Proposing Proactive Routing Protocols

The main reason to intend proactive routing protocols is their proactive route selection strategy (pre-determined route establishment) from each node to every other node immediately after the deployment of nodes in IoT-UWSN. These routes are ready to use instantly. In addition, these propose proactive routing protocols periodically update their routing table. These mechanisms help the IoT-UWSN in E2E delay minimization. However, they dissipate high EC. In addition, bandwidth and power management ratio in proactive routing protocols is higher than reactive and hybrid routing protocols [25].

### 1.6. Uniqueness of Proposed Protocols from Existing Protocols

In this paper, two routing protocols are proposed. However, they are different from the existing as follows:

1. In EP-VIR-Three, the forwarder node is selected with a minimum number of neighbor nodes to avoid collision and interference (using 3-hop neighbor’s information and cost function), whereas, in LMPC, the number of collisions are bit high.
2. In BF-SPF-Three, Bellman Ford algorithm is used for data routing with minimum EC. In addition, it solves the problem of routing loop problem. However, in LETR and LMPC, the EC is high due to certain DAs and collisions.
3. In LMPC, the binary tree generation starts from the source node. However, in the proposed protocol BF-SPR-Three, a binary tree is generated from the cross-node (node existing near the layers or coinciding with the layer). In this proposed protocol, the current forwarder first checks the 3-hop neighbor’s information of the forwarder node to avoid a void hole problem and then forward the DP.
4. Above discussed strategies result in the reliability of data transmission with minimum EC and E2E delay than the existing state-of-the-art routing protocols.

Abbreviations and acronyms for this paper are placed at the end of this paper, whereas, the rest of the paper is organized as follows: the problem statement of the proposed work is stated in Section 2. Detailed description of the existing protocols is given in Section 3. Section 4 presents the system model and description of the proposed protocols for deep understanding. Feasible regions using linear programming are computed in Section 5. Simulation results and discussion are presented in Section 6. Finally, this study is concluded in Section 7 with future directions.

## 2. Problem Statement

In IoT-UWSN, every sensor node has limited battery lifespan. Therefore, in IoT-UWSN, effective EC and reliable data delivery are the key concerns. A huge amount of energy is dissipated in void hole recovery (when a current source node does not find the next forwarder node in its transmission range) during the packets transmission. Many routing protocols are designed to address these issues. However, there still exists some chances of void hole occurrence, i.e., in [26]. In [26], a void hole recovery mechanism is presented. In addition, packet drop ratio is minimized to some extent. However, the long path establishment during the sparse network was a key challenge. The probability of the longer path establishment directly relates to nodes density. To address this issue, a Co-Improved Hydrocast protocol is proposed in [27]. The proposed protocol reduces the number of transmissions in the sparse region via opportunistic cooperation routing (by deploying some fixed nodes). This strategy enhances the network throughput using the short recovery path. However, the next hop forwarder node selection rises the problem of local optimal solution (in which the current source node considers the higher pressure level node as the next forwarder node instead of the low-pressure level node) and backward transmission.

**Figure 2:**
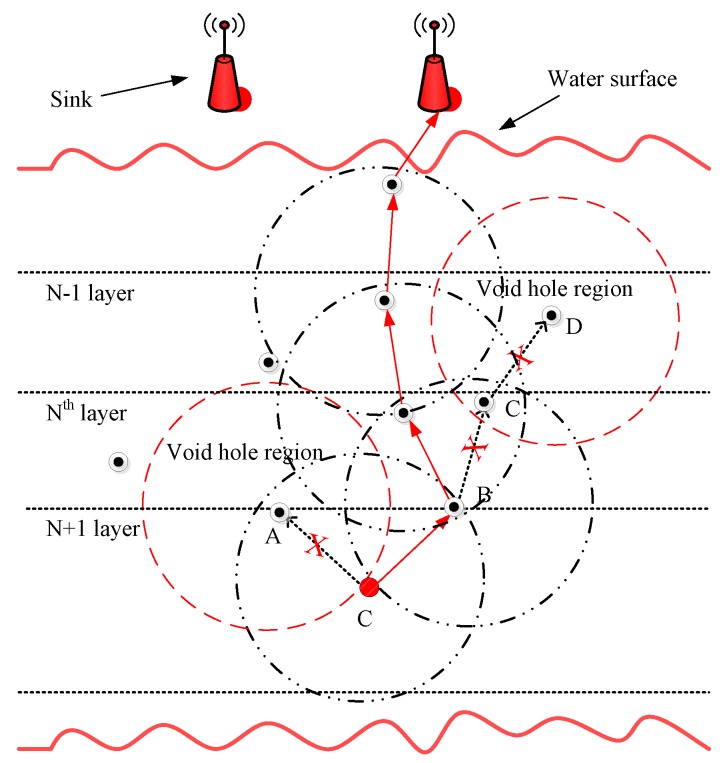
Forwarder node selection in EP-VIR-Three and BF-SPR-Three.

The above analysis shows that the selection of the next forwarder node can be further optimized to improve the performance of IoT-UWSN. Therefore, to reduce the probability of local optimal solution and backward transmission, two proactive routing protocols are proposed. The proposed protocols provide an energy effective path and interference-free transmission by avoiding the void hole problem (see Figure 2) and immutable forwarder node selection.

## 3. Detailed Description of the Existing Protocols

In this subsection, system models of the existing protocols, i.e., Hydrocast, Co-Improved Hydrocast, LETR, GEDAR and LMPC are discussed. The brief explanation and pictorial description of these routing protocols are presented in the following subsections.

### 3.1. Hydrocast

**Figure 3:**
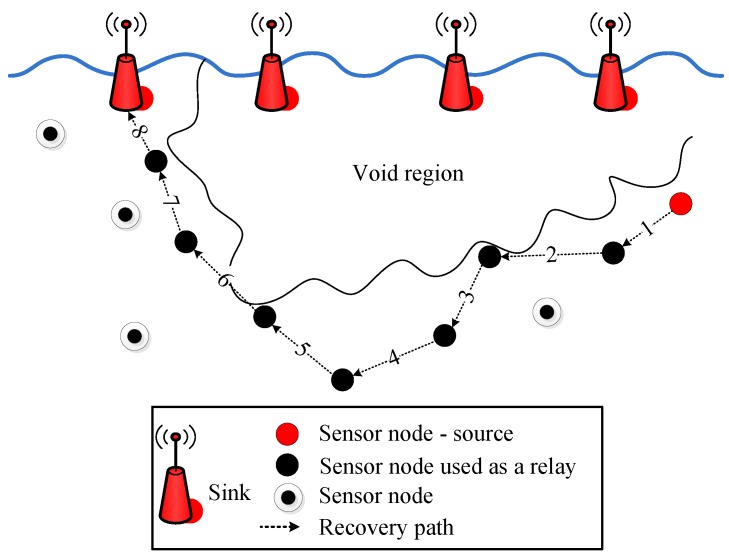
System model of hydrocast protocol.

In this routing protocol, a recovery mechanism is proposed to tackle the void hole problem. Moreover, the next forwarder node is elected on the base of its depth difference. The priority of a node for next forwarder node will be high, if its depth is lower than its neighbor nodes. This forwarder node selection minimizes the packet drop ratio and shows high performance during a dense network. However, in the sparse network, this strategy leads towards long recovery path as in Figure 3. This figure highlights the issue of the long path (during a sparse network). For example, the source node sends the DP towards sink via relay nodes (by avoiding the void hole region). This strategy results in longer recovery paths, i.e., (from node I to node VII). The probability of a longer recovery path increases during sparse network. Due to the aforementioned reasons, the network faces high E2E delay and high EC. This analysis shows that this protocol needs to be enhanced. Therefore, Co-Improved Hydrocast protocol is proposed. The detailed description of this protocol is presented in the next subsection.

### 3.2. Co-Improved Hydrocast

In Co-Improved Hydrocast, the aforementioned problem is tackled by the diminishing of transmissions (via opportunistic routing cooperation technique). The working of the routing protocol is two folded, i.e., in the first fold, some fixed sensor nodes are conveyed in the UWSN (at vital areas) and the remaining nodes are deployed independently. In the second fold, the idea of opportunistic routing is implemented to upgrade the UWSN throughput. Every node finds the neighbor nodes in its transmission range to advance the data and compute the Euclidean distance with neighbors. The priority to advance the information is based on how a sensor node is close to a sink node. In the proposed work, void nodes are avoided in two distinctive ways, i.e., if a source node itself becomes a void node, then the protocol follows recovery mechanism and starts back transmission (by selecting high pressure nodes to advance the information towards sink); otherwise, the low-pressure node is elected as the next potential node. The number of fixed nodes and their deployment benefits are:

1. Twelve static nodes are conveyed in the UWSN to diminish the number of transmissions [27].
2. Four sets including three fixed nodes are conveyed to cover the maximum area of the UWSN [27].
3. These fixed nodes reduce the retransmission in the UWSN and increase the PDR with minimum E2E delay [27].

**Figure 4:**
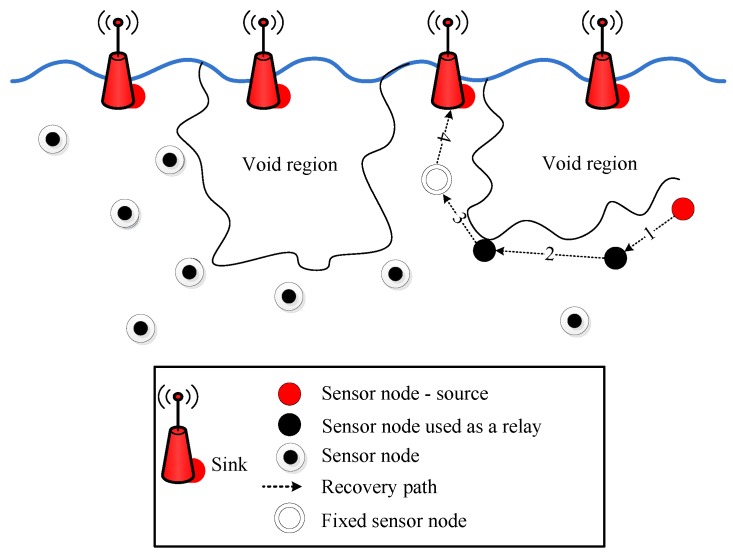
System model of co-improved hydrocast protocol.

**Figure 5:**
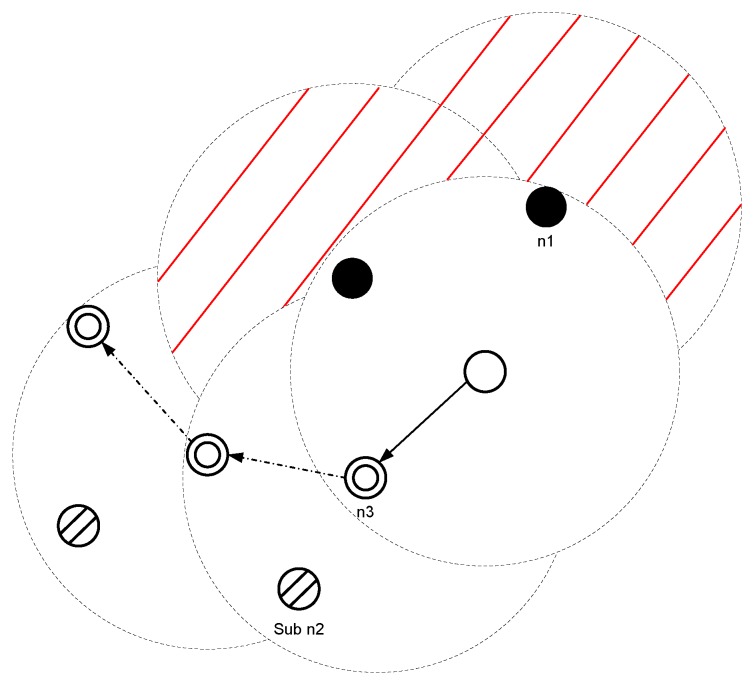
Back transmission and low pressure level nodes selection in co-improved hydrocast protocol.

This strategy enhances the network throughput using the short recovery path as in Figure 4. In this figure, the source node sends the DP to sink via relay nodes (by avoiding the void hole region) and fixed sensor nodes (deployed strategically). This strategy results in short recovery paths, i.e., (from node I to node IV). However, the next hop forwarder node selection raises the problem of local optimal solution (in which the current source node considers the higher pressure level node as the next forwarder node instead of the low-pressure level node) and back transmissions (see Figure 5). These aforementioned issues show that this protocol needs to be enhanced to minimize the EC and E2E delay by avoiding the void hole problem.

### 3.3. LETR

**Figure 6:**
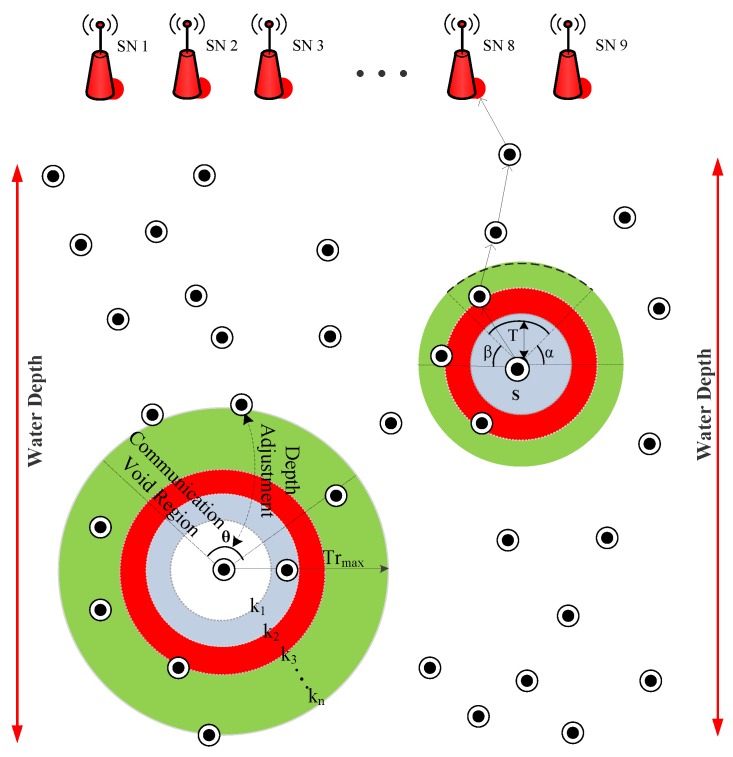
System model of lETR.

A homogeneous model is proposed in LETR as shown in Figure 6. It is clear from the figure that sensor nodes are randomly deployed in the network [14] and multiple sinks (housed with both acoustic and radio modems) are placed at the sea surface. These nodes are provided with GPS facility to determine their location. Initially, all sensor nodes broadcast the beacon message, while the sink nodes only once. The size of this beacon message is kept small (to minimize the EC). With the passage of time, the location of the nodes get updated and become ineffective. Therefore, sensor nodes broadcast periodic beacon (to be up to date). LETR prioritizes adaptive transmission range adjustment over DA to save the extra EC. It performs geographic and opportunistic routing by incorporating the location error with DA and transmission range adjustment. In the end, data reaches successfully at the sink node. We assumed that, if a DP is successfully received at one sink, it means that data is successfully delivered to the control station. The working of the protocol is as follows:

1. In LETR, every node performs periodic beaconing (which is basically broadcasted). However, each sink node broadcasts a single beacon message.
2. This message is broadcasted to remain up to date with neighbor node information (the neighbor nodes are selected using angle based neighbor selection [14]) lies in its vicinity.
3. Transmission power of the nodes is divided into *k* different levels.
4. This division helps the nodes during void hole recovery.
5. When a node does not receive a beacon, it means that it is declared as a void node. Therefore, it adaptively adjusts their transmission range (step I).
6. After DA, each sensor node broadcasts the beacon message once again. This beacon message includes relative information of their neighbor nodes existing in its vicinity, i.e., location information and current clock time in beacon message (to identify the recent beacon from a node).
7. If a node does not find the eligible forwarder node in its first stage (after step I), then node adjust their transmission power and continue the process to eliminate the void node (step II).
8. Then, the current source node finds the potential node existing at that stage (after step II) and sends a packet of acknowledgment including coordinates of the node.
9. The DA is rare in LETR. Therefore, the beaconing mechanism is used rarely.
10. This process remains continuous until all the data reaches the destination.

The detailed description of this protocol with the algorithm is given in [14].

### 3.4. GEDAR

In this subsection, GEDAR protocol is discussed with its pictorial description [23]. GEDAR performs geo opportunistic routing with DA. Wherever a sensor node discovers a void node in its range, it adaptively amends its depth. Afterward, it again discovers its neighbor nodes in its range. If it found the next potential node, it advances the DP; otherwise, it again amends its depth. However, continuous DA leads the UWSN towards an energy hole. This energy hole dissipates high EC. At this end, high EC results in maximum energy depletion, which results in a void hole. If the cost of a mobile node is somehow reduced, the high E2E delay affects the network performance. The induction of void node in the UWSN increases backward transmissions, which also increases the E2E delay. These aforementioned problems encourage the researchers to enhance this protocol.

**Figure 7:**
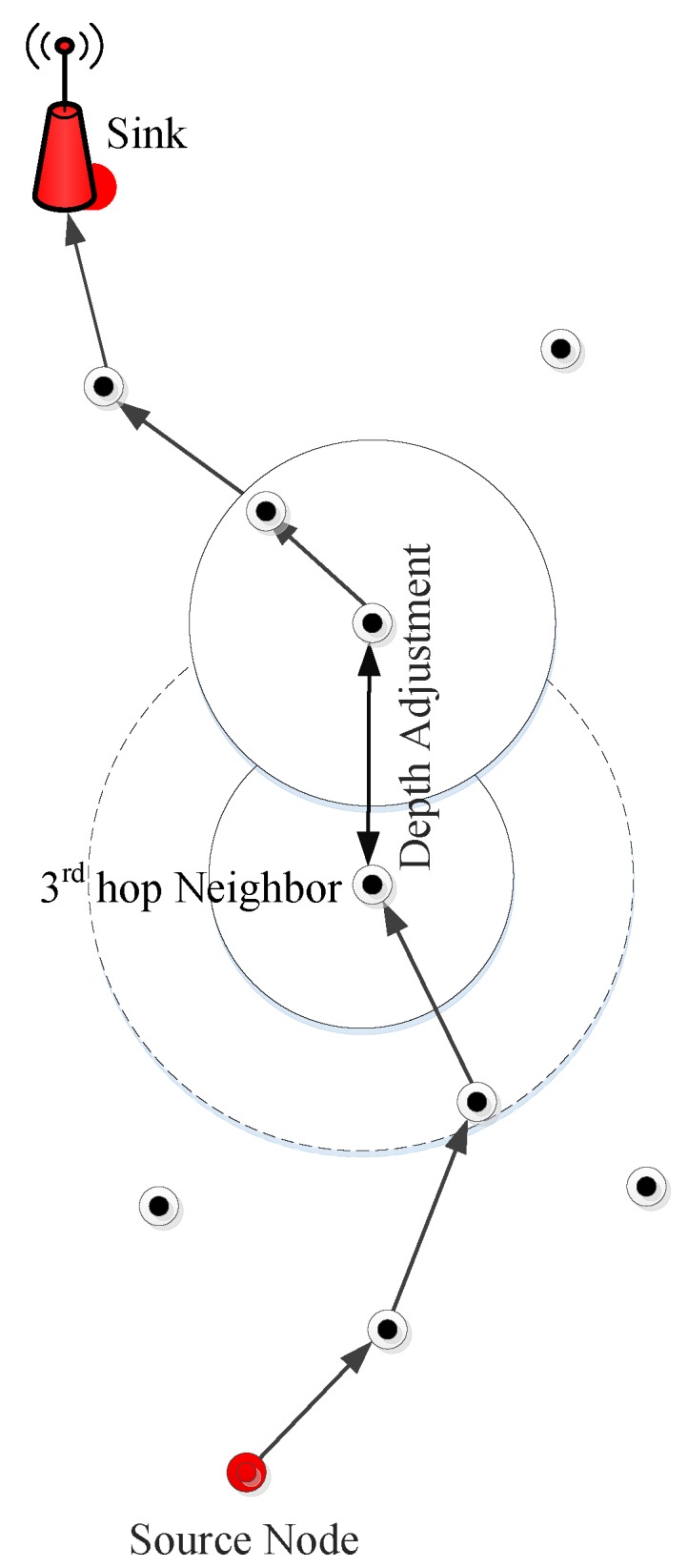
DA in GEDAR.

The DA in GEDAR is shown in Figure 7. In this figure, the source node transmits the DP to its forwarder node, but, at its 3*^rd^* hop, the respective node has no next potential forwarder node. Therefore, this node needs to adjust its depth to find the next forwarder neighbor node (to forward its DP). After its DA, this node successfully forwards the data towards the sink node (using multi-hop transmission).

### 3.5. LMPC

**Figure 8:**
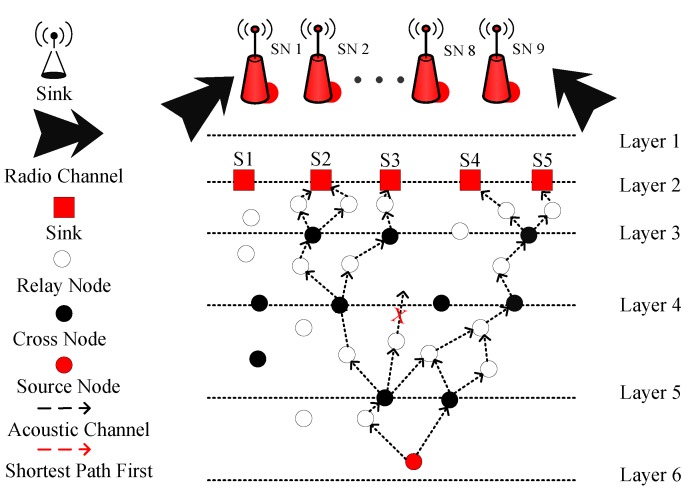
System model of LMPC.

In LMPC, the layered multi path routing approach is used. In this protocol, layers are dividing the network in such a way that layers get closer near the sea surface. This happens to minimize the noise (i.e., ships noise) effect on the sea surface. The network of LMPC consists of the cross (the node on the layer and just coinciding with the layers), normal (simple sensor nodes), source and sink (sonobuoy) nodes with the surface gateways and channel pathways. These sonobuoys act as an embedded system in an underwater environment. The binary tree formation is the main feature of this protocol. This binary tree is generated from the source node as well as from the cross nodes. Multiple copies of data are originated to make reliable data transmission. However, multiple transmission causes several packets to collide. As a result, the energy of the network is dissipated fast and network collapse with the passage of time. The network architecture of LMPC is shown in Figure 8. In this figure, the source node sends multiple copies of data to its forwarder nodes. When these copies reach the next forwarder node, the protocol checks the type of node. If the node is a simple node, it unicasts the DP towards its potential forwarder node; otherwise, it generates a binary tree and sends multiple copies of data to its next forwarder nodes (here, multiple copies means two copies). This process continues until all the data reaches the destined sink.

## 4. System Model and Description of the Proposed Protocols

This section includes the system model of the proposed protocols in detail. In this work, two proactive routing protocols, namely: EP-VIR-Three and BF-SPR are proposed to remove the problem of void hole in IoT-UWSN. The protocol EP-VIR-Three discovers the complete route by selecting the next potential forwarder by checking the 3-hop neighbor’s availability of the next forwarder node. EP-VIR-Three selects those forwarder nodes which have minimum neighbor nodes. The protocol BF-SPR-Three minimizes the void hole probability using proactive and efficient path selection (using ‘Bellman–Ford’ (BF) algorithm) with 3-hop neighbor’s information. The detailed discussion is given below.

### 4.1. Proposed Protocol 1 (EP-VIR-Three)

**Figure 9:**
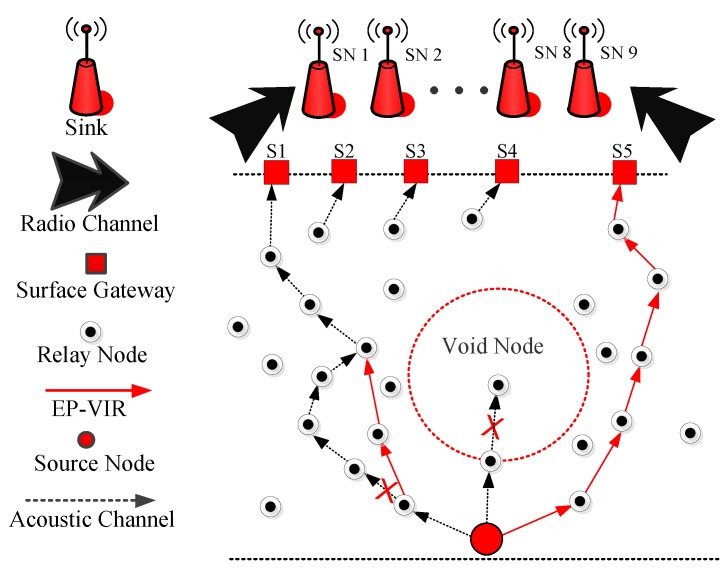
System Model of EP-VIR-Three.

In this subsection, the network architecture of EP-VIR-Three is discussed in detail (see Figure 9). Then, detailed theoretical analysis (including propagation and data transmission) is presented. In the end, the steps for EP-VIR-Three working are given in detail (see Algorithm 1).

#### 4.1.1. Network Configuration

In the proposed protocol, the network model is housed with multiple sinks (nine sinks) and relay nodes varying from (150–450) [27]. The responsibility of these nodes is to collect and forward the DPs towards the sink by collecting their own DPs and from their current forwarder nodes. The multiple sinks are placed on the water surface and these sinks are assumed to be stationary. These sinks are housed with both radio and acoustic modem. The radio modem is used for the communication among sinks and base stations, while the acoustic modem is used for underwater communication and data collection among sinks and underwater sensor nodes (using multi-hop). Moreover, we assumed that:

1. Firstly, sinks have high energy. Moreover, they are connected with each other to balance the load of DPs on respective sinks.
2. Secondly, if the data is successfully delivered from any of the relay nodes to any of the sink, then it means that data is successfully delivered to the control station.
3. In the end, we have not considered the movement of nodes in a vertical direction, which is almost negligible, whereas, we have only considered the mobility of the nodes in the horizontal direction (due to water currents).

#### 4.1.2. Detailed Theoretical Analysis of Propagation and Data Transmission Model

In this routing protocol, every node broadcasts a message to find its neighbor nodes and a number of hops. These two parameters are computed through beacon from the sink. Furthermore, the reactive beaconing mechanism is adapted to synchronize the routing table of the sensor nodes. This information is shared with neighbors by using hello packet (see Table 2). When a sensor node receives a hello packet, it upgrades its routing table (see Table 3). When a sender sends DP, it includes a hello packet. Upon data reception, the receiver node upgrades its table again, if its depth is higher than sender node [7].

EP-VIR-Three selects the next forwarder node by checking the 3-hop neighbor’s availability of the next forwarder node to avoid the void hole occurrence and data loss. For example, in Figure 2, the current source node *C* checks its neighbor nodes and select the current forwarder node by checking the 3-hop neighbor’s availability of the next forwarder nodes (i.e., node *A* and node *B*). Therefore, node *C* will select the node *B* as its current forwarder node because node *B* has neighbors until its 3*^rd^* hop. Furthermore, we exploited the 2D mobility model and piggybacking mechanism to lower neighbors’ request.

The source node selects the next forwarder node on the bases of Cost Function (CF) value, which is calculated using the Equation (1) as in [7]:

(1) $$\;CF\left(k\right)=\frac{Dis(j,k)}{Hop(k)\times Neighbor(k)},$$

where Hop(k) is the *k*th forwarder node of the source node, Neighbor(k) is used to represent the neighbors of the *k*th node and Dis(j,k) is used to represent the distance between the *k*th forwarder node and the source node *j*. This equation shows that CF has the maximum value until the forwarder node has the minimum number of neighbors, minimum hops from the sink and the maximum distance from the sender node (source node). The node having maximum CF value and 3-hop neighbors is selected as next forwarder for the current source node. Then, the source node broadcasts this information to its neighbors using beacon message. When the neighbors receive this beacon message, they perform a comparative analysis of their nodes ID. If this ID matches the ID of the DP, then this node is selected as a forwarder. In addition, all other nodes discard that DP. This process continues until data reaches the destined sink. Then, the sink forwards the data to a respective control station.

How to ensure minimum neighbors? Protocol EP-VIR-Three selects the next potential node using 3-hop neighbor’s information, its distance and its hop count from destined sink node. Therefore, the potential forwarder node is elected on the base closeness from the destined sink. The reason behind this selection criteria is maximum EC during communication between the nodes. Therefore, protocol chooses the path with the minimum participating nodes in the communication range. In the current scenario, this selection criteria affirms minimum neighbor nodes. The current source node elects the next potential forwarder node using these parameters, i.e., Hop(k), Neighbor(k) and Dis(j,k).

How 3*^rd^* hop neighbors availability acquired? In Figure 2, forwarder node selection criteria for both proposed protocols is pictorially described. In this figure, the current source node *C* checks its neighbor nodes and selects the current forwarder node by checking the 3-hop neighbor’s availability of the next forwarder nodes (i.e., node *A* and node *B*). Therefore, node *C* will select the node *B* as its current forwarder node because node *B* has neighbors until its 3*^rd^* hop.

#### 4.1.3. Description of the Algorithm 1

In this subsection, the data forwarding algorithm for EP-VIR-Three is discussed. Firstly, the input parameters for the routing protocols are initialized. Then, the sensor, sink and relay nodes are deployed in the network with different surface gateways. Afterwards, a beacon message is broadcasted, which helps the neighbor nodes to update their routing table. Moreover, the protocol checks the 3-hop neighbors availability to avoid the void hole problem. In the end, transmission begins by selecting the next forwarder node after confirming 3-hop neighbors availability with a minimum number of neighbors. Furthermore, if the current forwarder lies within the range of the respective sink, then the data is directly transmitted to the destined sink; otherwise, the next forwarder node is selected (provided by ‘CF’ value, 3-hop neighbors information and its depth should be greater than the sender node).

**Algorithm 1** Algorithm of EP-VIR-Three for data forwarding

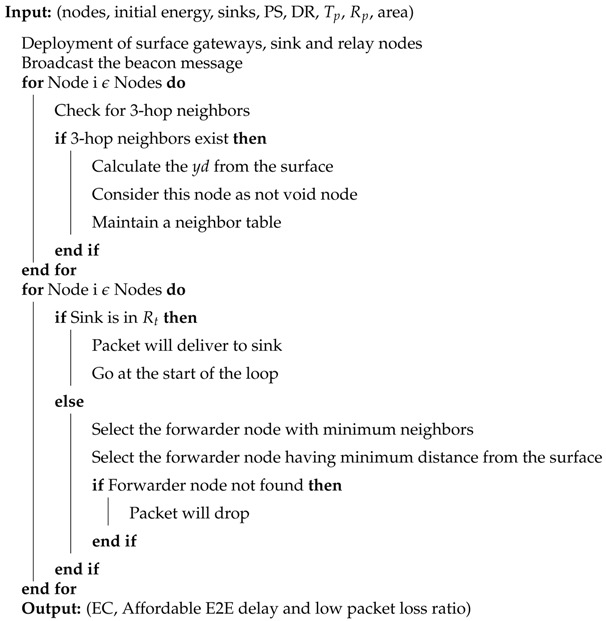



### 4.2. Proposed Protocol 2 (BF-SPR-Three)

This subsection discusses the network architecture, propagation and energy model of BF-SPR-Three. Afterward, the neighbor’s selection procedure and tree establishment criteria are elaborated. Then the steps included in BF-SPR-Three are shown in Algorithm 2.

#### 4.2.1. Network Configuration

Before explaining the network architecture of BF-SPR-Three, a few terms need to be defined here. The nodes which perform data forwarding including their own sensed data and the data they received from higher depth nodes, are known as relay nodes and the nodes which lie on the layer or coinciding with the layers are considered as cross nodes.

The proposed protocol BF-SPR-Three consists of sinks, relay nodes, cross nodes varying from (150–450) and surface gateways. Basically, the sink works as an embedded unit and housed with both acoustic and radio modems. The radio modem is used for the communication among sinks and base stations, while the acoustic modem is used for underwater communication and data collection among sinks and underwater sensor nodes (using multi-hop). Moreover, we assumed that:

1. Firstly, sinks have high energy. Moreover, they are connected with each other to balance the load of DPs on respective sinks.
2. Secondly, if the data is successfully delivered from any of the relay nodes to any of the sinks, then it means that data is successfully delivered to the control station.
3. In the end, we have not considered the movement of nodes in a vertical direction, which is almost negligible, whereas we have only considered the mobility of the nodes in the horizontal direction (due to water currents).

Initially, all the sensor nodes are randomly distributed in the underwater network and they are in sleeping mode (in this mode, nodes are basically alive, consuming a negligible amount of energy; however, they are not participating in the transmission process). When a node (during sleeping mode) receives a DP, then this node changes its status from sleeping to the active mode (in this mode, nodes actively participate in the transmission process and consume energy during packets reception and transmission). If any node is not participating in the transmission process, then that nodes get back to the sleeping mode again. In addition, the key step in the proposed protocol is binary tree generation. This tree generation procedure is adopted for multi-path transmission. Let us consider the LMPC routing protocol scenario (as shown in Figure 8). In LMPC, the UWSN is distributed into layers. Moreover, the binary tree generation helps the routing protocol in reliable data delivery. Then, from every sensor node, different copies of DP are transmitted. This process remains continuous until the DP reaches the destined sink. While forwarding the DP from sea depth to lower depth, different noises including shipping noises add up with data and produce bit error in the packets. If this hindrance exceeds a certain limit, it ultimately drops the DP. Due to this reason, multiple copies of DP are required for reliable data delivery.

**Figure 10:**
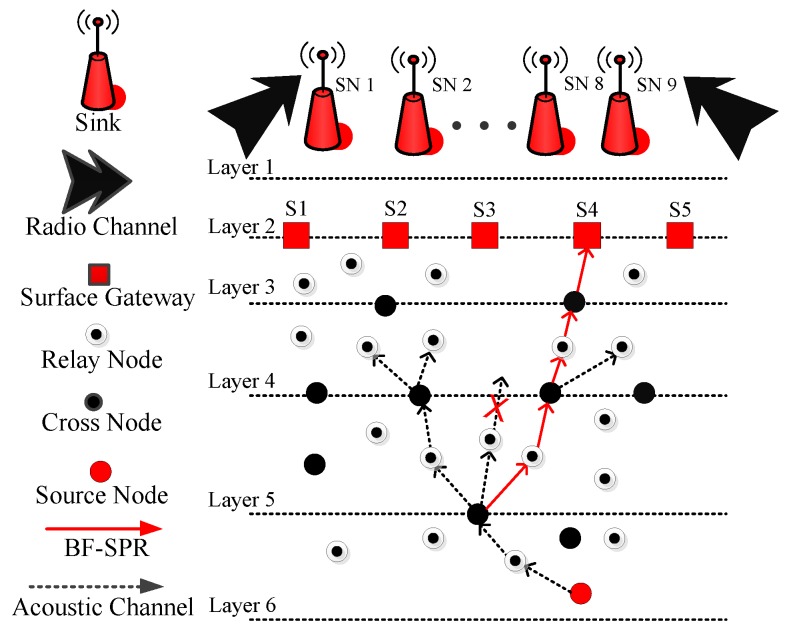
System Model of BF-SPR-Three.

By keeping these parameters in mind, we have implemented a new routing protocol named BF-SPR-Three. The beacon mechanism is similar for EP-VIR-Three routing protocol. In BF-SPR-Three, the complete shortest and the efficient path is computed using the ‘BF’ algorithm. The network environment is distributed into unequal layers (closer near the sea surface and distanced at the sea bottom (see Figure 10)). In the current protocol, the sensor node (source) generates a single copy of the data. As the data is advanced to the lower depth layer using relay nodes (selected from the BF algorithm), then the current forwarder (source node) checks the next forwarder node (destination node) regarding whether it is a cross-node or a simple relay node. Upon DPs reception, if the next forwarder node is a relay node, then it unicasts the DP. On the contrary, a cross-node uses IP multicasting technology (a bandwidth preserving technology that minimizes the traffic of DPs by instantaneously delivering a single stream of data to potentially thousands of corporate receivers) to transmit the DP. In current work, the IP of the neighbor nodes is their unique ID. In the end, these DPs are directed towards surface gateways and ultimately data reaches the sink. DPs are combined at the destined sink to generate the real DP and advanced towards control station.

Why ‘BF’ Algorithm?

In graph theory, negative weight edges can make negative weight cycles, i.e., a cycle which will diminish the path by returning to a similar point. Briefest path algorithms, i.e., ‘Dijkstra’ Algorithm, are not ready to distinguish such a cycle. Eventually, they give an unseemly outcome since they can experience a negative weight cycle and reduce the route length. Afterward, in this work, we chose to utilize a ‘BF’ algorithm for our proposed protocol.

Functioning of ‘BF’ Algorithm?

‘BF’ algorithm works by overestimating the length of the route from the earliest starting vertex to all unique vertices. By then, it iteratively slackens up those evaluations by finding new routes that are shorter than the as of late overestimated ways. By doing this on and on for all vertices, we can guarantee that the final route is upgraded. The essential steps of the ‘BF’ algorithm are:

1. Starts with a graph having weights,
2. Selects a starting node and allocates the infinity path value to all other nodes,
3. Visit each vertex and relax the path if it inaccurate,
4. Iterate these steps up to the number of nodes in the vertices,
5. After all the nodes have their path lengths, the algorithm will check for the negative cycles.

#### 4.2.2. Detailed Theoretical Analysis of Propagation and Data Transmission Model

In this section, the propagation model and the EC model of the acoustic signals are explained. In addition, the absorption constraints are also formulated in the underwater environment. The propagation and EC model include:

**Channel Fading Model in BF-SPR-Three**

The attenuation of the acoustic channel is formulated using Equation (2) as in [24]:

(2) $$\;A\left(Dis,Feq\right)=Dis^{k}\times \alpha \left(Feq^{Dis}\right),$$

where *k* represents the spreading factor. Whereas, $\alpha (Feq^{Dis})$ denotes absorption coefficient. Basically, k is describing the propagation pattern. For cylindrical spreading, *k* is taken as 1 with limited propagation pattern (with no attenuation). Similarly, for practical spreading, the value of *k* is taken as 1.5. In this spreading, the signal propagation considers both transmission and attenuation losses to get the precise result. In the end, *k* is taken as 2 for spherical spreading. In spherical spreading, the signal propagates in omni directions. However, these waves move a long way from source by keeping the power of the signal same. The α(Feq) is defined by Thorp’s Model as in [24].

(3) $$\alpha \left(Feq\right)=\frac{0.11\times Feq^{2}}{1+Feq^{2}}+\frac{44\times Feq^{2}}{4100+Feq^{2}}+2.75\times 10^{-4}\times Feq^{2}+0.003.$$

**Figure 11:**
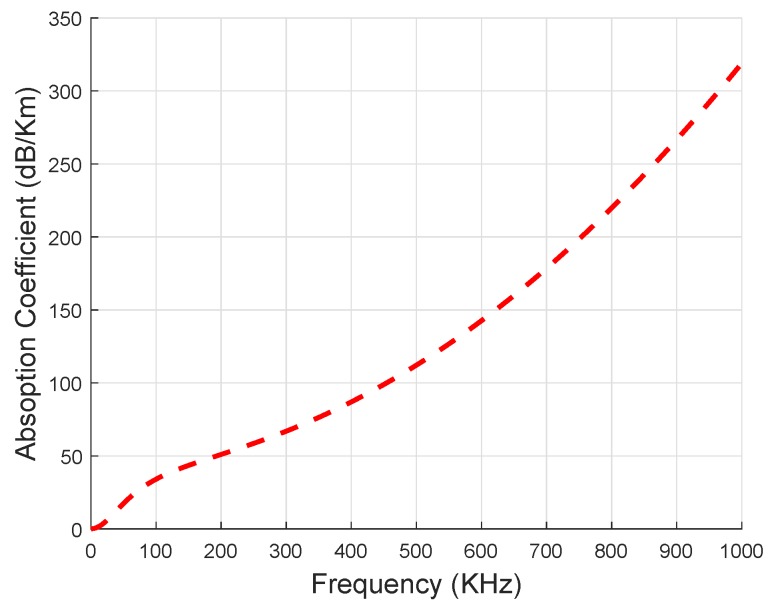
Absorption and frequency relation in the proposed protocols.

Here, α(Feq) is measured in dB/Km and *f* is measured in KHz, respectively. The relationship between α(Feq) and Feq is shown in Figure 11. From the figure, it is obvious that the absorption rate of the acoustic signal increases with the increase in Feq value. IoT-UWSN includes shipping noise, thermal noise and turbulence noise. Therefore, these noises affect the acoustic channel during transmission. The total Noise Power (NP) including the spectral density of all the noises at frequency Feq is calculated as [24]:

(4) $$\;NP\left(Feq\right)=\sum_{i=1}^{tn}NP_{i}\left(Feq\right),$$

where, the aforemention total noises are represented by tn. The value of NP is measured in dB. The attenuation over the distance Dis is calculated using Equation (5).

(5) $$NP^{Dis}\left(Feq\right)=\sum_{i=1}^{tn}\frac{NP_{i}\left(Feq\right)}{Dis^{k}\alpha \left(Feq^{Dis}\right)}.$$

Here, $NP^{Dis}\left(Feq\right)$ is measured in dB and it should be lies in between (1-tn).

**Channel Capacity Model in BF-SPR-Three**

The Channel Capacity (cc) of the acoustic channel is formulated using Equations (6) and (7) as in [24].

(6) $$\;cc=B\cdot \log_{2}\left(1+SNR\right),$$

(7) $$\;AC\left(PER\right)=\frac{cc}{1-H_{2}\left(PER\right)},$$

here, *B* represents the bandwidth of the acoustic channel, SNR is used to denote signal to noise ratio (calculated using Equation (8)). Meanwhile, PER is used to represent the number of bits without any error and $H_{2}$ is a binary entropy function using PER, which is defined as $H_{2}=\left(PER-1\right)\log_{2}\left(1-PER\right)\left(PER\log_{2}PER\right)$.

(8) $$\;SNR=\frac{P/A(Dis,Feq)}{NP^{Dis}\left(Feq\right)},$$

here, *P* is the power of the acoustic signal and Dis is the distance among n−1 and *n*th nodes. Whereas, SNR follows the additive white gaussian noise channel.

**Transmission and Receiving Energy Calculations in BF-SPR-Three**

The EC of the sensor nodes during the packet transmission and reception is calculated using Equations (9) and (10).

(9) $$\;E_{trans}=\frac{T_{p}\times PL}{DR}\times Dis,$$

(10) $$\;E_{rec}=\frac{R_{p}\times PL}{DR}\times Dis.$$

Here, the EC of the sensor nodes during the packet transmission is denoted as $E_{trans}$. $E_{rec}$ is used to represent the EC of the sensor nodes during the packet reception. Whereas, $T_{p}$ and $R_{p}$ denotes transmission and reception power of the sensor node, respectively. Packet size is represented as PS and DR is used to represent the data rate of the acoustic channel. The total residual energy of the nodes in the underwater environment after forwarding all the DP is denoted as $E_{trans}^{rem}$ and computed using Equation (11).

(11) $$\;E_{trans}^{rem}=\sum_{DP=1}^{N}\sum_{HC=1}^{THC}n_{i}-E_{trans}.$$

Here, *N* represents the total number of DPs in the network. Where total hop counts (for a single DP from source to the destination) are denoted as THC. Whereas, $n_{i}$ is used to represent ith node. The total residual energy of the nodes in the underwater environment after receiving all the (DPs) is denoted as $E_{rec}^{rem}$ and computed using Equation (12).

(12) $$\;E_{rec}^{rem}=\sum_{DP=1}^{N}\sum_{HC=1}^{THC}n_{i}-E_{rec}.$$

The initial energy of the node is represented as $E_{i}$. Whereas, the total energy of the network is denoted as $T_{E}$ and calculated using Equation (13).

(13) $$\;T_{E}=E_{i}-\left(E_{trans}^{rem}+E_{rec}^{rem}\right).$$

#### 4.2.3. Layers Division in BF-SPR-Three

In BF-SPR-Three network is distributed into sublayers for reliable data delivery. As the noise rate increases while moving from higher depth area towards the lower depth area. Therefore, in this protocol, the UWSN is distributed into unequal layers. These layers become closer near the sea surface and far in the sea depth. The reason for being closer is the high noise ratio at sea surface. This happens to reduce high noise ratio at the sea surface. The basic purpose of this approach is reliable data delivery and minimum packet drop ratio. Therefore, the ratio for the unequal layers distribution is computed by Equation (14).

(14) $$\;C\left(i,j\right)=\frac{k_{1}i_{2}+k_{2}i_{1}}{k_{1}+k_{2}},\frac{k_{1}j_{2}+k_{2}j_{1}}{k_{1}+k_{2}}.$$

**Figure 12:**
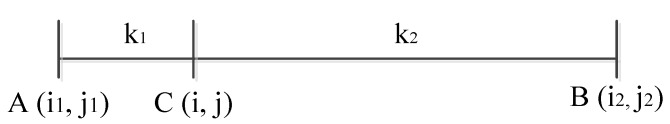
Layer Division.

Here, the endpoint coordinates are denoted by $i_{1},j_{1},i_{2}$ and $j_{2}$, whereas C(i,j) is the division point (see Figure 12). Whereas, the point *C* divides the line in $k_{1}:k_{2}$ depending upon the strength of noise (using Equation (8)).

#### 4.2.4. Description of Algorithm 2

In this subsection, the data forwarding algorithm for the proposed protocol (BF-SPR-Three) is discussed. The detail is given below.

**Algorithm 2** Algorithm of BF-SPR-Three for data forwarding

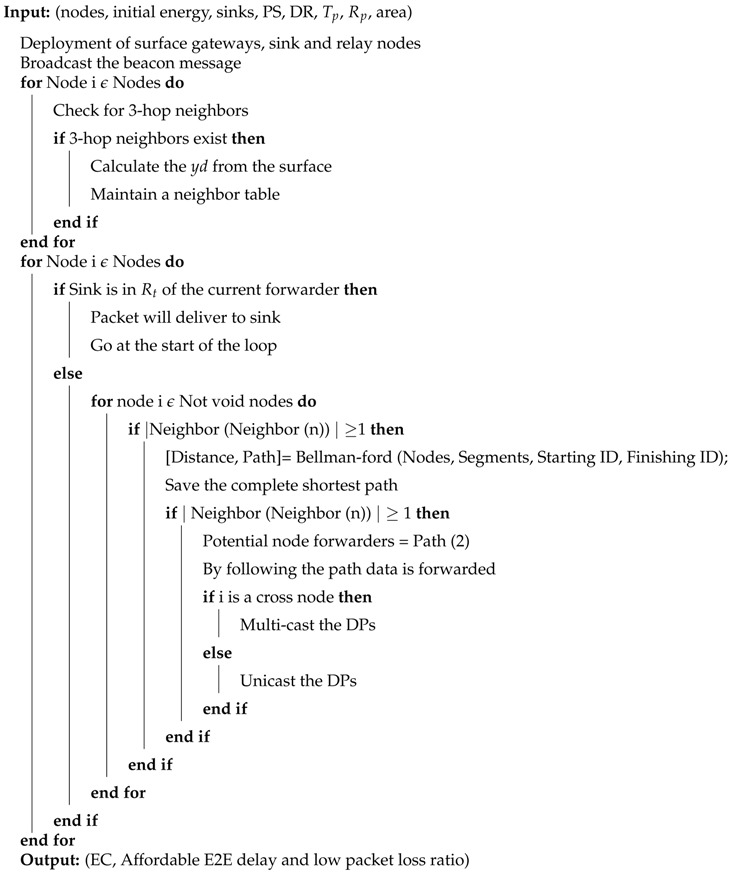



Firstly, the input parameters for the routing protocols are initialized. Then, the sensor, sink and relay nodes are deployed in the network with different surface gateways. Afterwards, a beacon message is broadcasted, which helps the neighbor nodes to update their routing table. Moreover, the protocol checks the 3-hop neighbor’s availability to avoid the void hole problem. In the end, transmission begins by selecting the next forwarder node given by the ‘BF’ algorithm (by providing the fastest and shortest path, to route the DP from the source node to the destined sink). Moreover, if the next forwarder is the simple sensor node, it will unicast the DP; otherwise, it will multicast the DP. In the end, if this forwarder node lies within the range of respective sink, then the data reaches the destined sink (using a binary tree generation approach); otherwise, the next forwarder node is selected (provided by the ‘BF’ algorithm).

#### 4.2.5. How Are Neighbors Selected in BF-SPR-Three?

In BF-SPR-Three protocol, the neighbors selection is similar to EP-VIR-Three. Whereas, distance from source to destined node is computed using Euclidean Equation (15):

(15) $$\;Distance_{\left(i,j\right)}=\sqrt{{\left(i_{1}-i_{2}\right)}^{2}+{\left(j_{1}-j_{2}\right)}^{2}},$$

whereas $\left(i_{1},i_{2}\right)$ and $\left(j_{1},j_{2}\right)$ are the coordinates of source and receiver node. Coordinates (i,j) are obtained from the beacon message and depth (yd) is obtained by calculating the depth of the node from the sea surface. The value of yd is used to find that the next forwarder node lies closer to the sink. The following constraints must be satisfied to find the optimum forwarders:

1. $Distance_{\left(i,j\right)}$ < Transmission Range $(R_{t}),$
2. Depth of the current forwarder should be greater than the next elected forwarder node.

#### 4.2.6. Binary Tree Generation in BF-SPR-Three

One of the following two scenarios must be true during packets transmission:

1. A sensor node is used for linear transmission.
2. There must be a cross-node for generating a binary tree.

If the node is a simple sensor node, it will unicast the signal. On the contrary, binary tree formation starts from the cross-node for multicasting (using IP broadcasting technology), in which every parent node has almost two child nodes.

## 5. Mathematical Formulation Based on Linear Programming

In this section, mathematical formulation is performed using linear programming that checks the feasibility of the proposed protocol (verified by performing the extensive simulations). To achieve the optimal solution for the objective function, we define some constraints. By using these constraints, the feasible regions for the network throughput and EC are drawn and their brief description is given below.

### Feasible Region for Energy Minimization in Proposed Protocols

The objective function to minimize the EC is given in Equation (16):

(16) $$Min\;\Sigma_{r=1}^{max}E_{consumption}\left(r\right)\;\forall \;r\in max,$$

whereas constraints for Equation (16) are as follows:

1. The $E_{trans}$ and $E_{rec}$ ≤ $E_{trans}^{rem},$
2. The $E_{trans}$ and $E_{rec}$ ≤ $E_{i},$
3. The DP should be transmitted within the $R_{t}$ of the node.

Total energy consumed is the sum of transmission and receiving energy of the nodes, i.e.,

(17) $$\Sigma_{r=1}^{max}E_{consumption}\left(r\right)=E_{trans}+E_{rec}\;\forall \;r\in max,$$

where

(18) $$E_{trans}=T_{p}\left(feq\right)\left(\frac{PS}{DR}\right).$$

The $E_{trans}$ ranges from (0.693–2.775) is used in the current work, where $T_{p}\left(feq\right)$ is representing the transmission power with frequency feq.

(19) $$E_{rec}=R_{p}\left(feq\right)\left(\frac{PS}{DR}\right).$$

**Figure 13:**
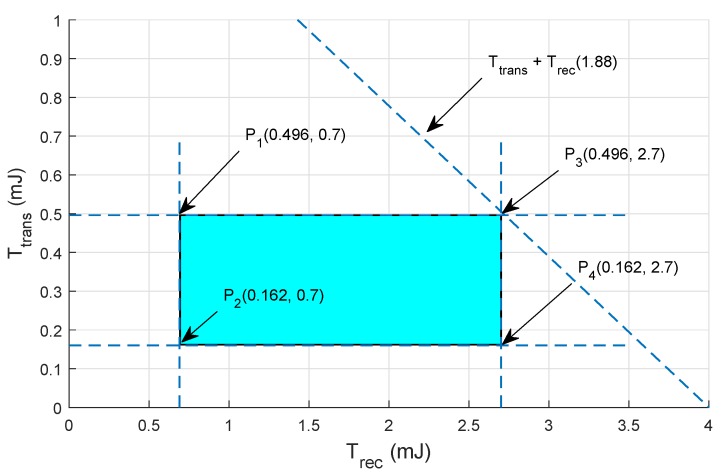
Feasible region for EC minimization in EP-VIR-Three.

**Figure 14:**
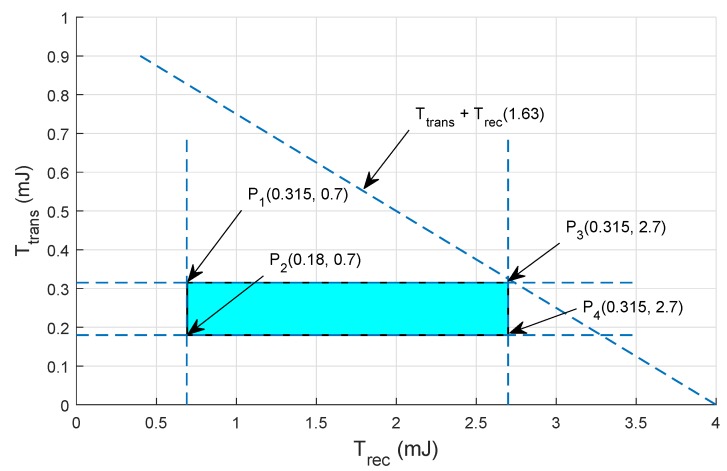
Feasible region for EC minimization in BF-SPR-Three.

The $E_{rec}$ ranges from (0.002–0.0087) is used in the current scenario, where $R_{p}\left(feq\right)$ is representing the receiving power with frequency feq. Feasible region for minimum EC are shown in Figure 13 and Figure 14.

The points on the feasible regions boundary in EP-VIR-Three are as follows:

$P_{1}\left(0.496,0.7\right)\;mJ,$
$P_{2}\left(0.162,0.7\right)\;mJ,$
$P_{3}\left(0.496,2.7\right)\;mJ,$
$P_{4}\left(0.162,2.7\right)\;mJ.$

The points on the feasible regions boundary in BF-SPR-Three are as follows:

$P_{1}\left(0.315,0.7\right)\;mJ,$
$P_{2}\left(0.18,0.7\right)\;mJ,$
$P_{3}\left(0.315,2.7\right)\;mJ,$
$P_{4}\left(0.315,2.7\right)\;mJ.$

## 6. Simulation Results and Discussion

For the evaluation of proposed protocols, different simulations have been performed in terms of DA, EC in the DA of the nodes and EC of the network during transmission of DPs with affordable E2E delay. The proposed protocols outperformed in alleviating the void holes and interference (experiences during packets transmission). Therefore, comparative analysis has been performed to validate the performance of the proposed protocols with other existing state-of-the-art proactive and reactive routing protocols.

In the next subsections, simulation parameters are discussed. Afterwards, the simulation results are presented and elaborated with detailed reasoning.

### 6.1. Simulation Parameters

**Figure 15:**
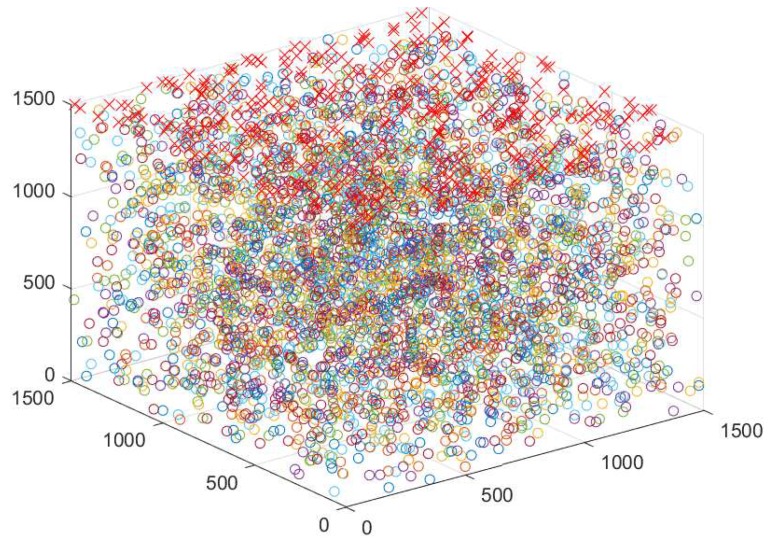
Nodes deployment.

In the simulation environment, nodes varying from (150–450) are randomly deployed in UWSN environment of dimensions 1500 m × 1500 m × 1500 m (see Figure 15) with nine sinks (at sea surface). We kept the transmission range, DR and PS of the DP as 250 m, 50 Kbps, and 200 bytes, respectively. The initial energy of the sensor nodes is kept 100 J, where the transmission and receiving the power of DP is kept at 2 W and 0.1 W. To handle the mobility of nodes, we have considered the node speed in the horizontal direction 2 m/s. Moreover, the propagation speed of the acoustic wave is kept 1500 m/s along with bandwidth of 4 kHz. Meanwhile, the size of beacon is kept 50 bits. We compared the existing work by increasing the node density to show the efficacy of the proposed work. The simulations are conducted in Aqua-Sim (NS-2-based UWSN simulator). The above-mentioned parameters are taken from [23] and listed in Table 4.

#### 6.1.1. EC

**Figure 16:**
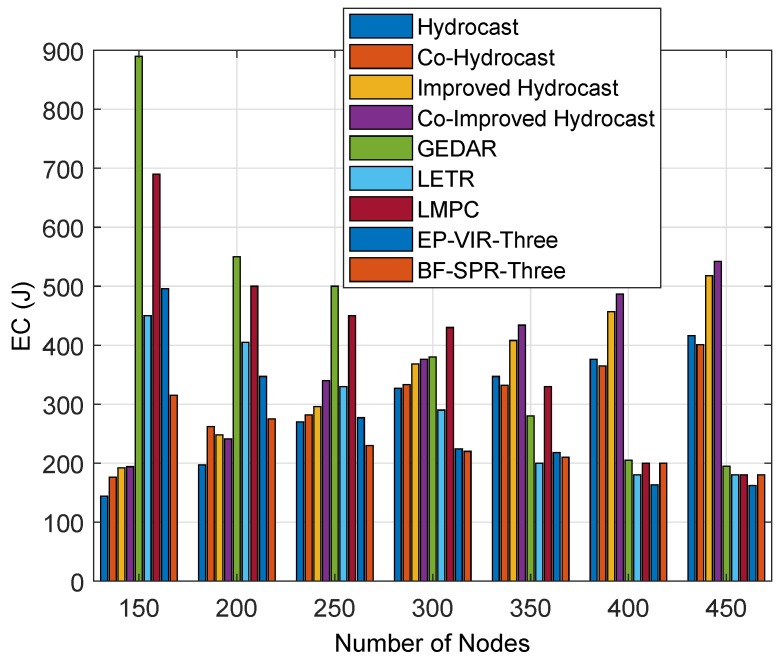
EC during packet transmission.

The EC of the proposed and benchmark protocols is shown in Figure 16. This figure shows the EC of the sensor nodes in IoT-UWSN (during packets transmission). It is obvious from the figure that GEDAR, LMPC and LETR have high EC until the network is sparse. The reason for high EC is that GEDAR performs several DAs for void hole alleviation. These DAs result in excessive EC in GEDAR. Meanwhile, LMPC has high EC due to multiple paths. This tree generation helps the IoT-UWSN to perform reliable data delivery. Similarly, in LETR, excessive DAs and adaptive transmission range adjustments result in high EC. In contrary, the EC reduces by increasing the nodes density (due to several alternative paths). Therefore, the EC of Improved Hydrocast is minimum because of short recovery paths for void nodes. Fixed deployment of the nodes also contributes towards minimum EC of the protocol. Whereas, Co-hydrocast and Co-Improved Hydrocast consume higher energy than Improved hydrocast routing protocol because of an opportunistic cooperation technique. At this end, the EC in Hydrocast in higher than both aforementioned protocols because of longer recovery paths.

In proposed protocols, EC is high when the UWSN is sparse. In contrary, EC is low. EP-VIR-Three provides the path using the potential forwarder node (by checking 3-hop neighbor’s availability) with minimum neighbors. These forwarders help in collision-free communication with minimum interference. Therefore, little energy is dissipated on 3-hop neighbors checking and during collision-free path selection (on the base of CF value), while, in BF-SPR-Three, the EC is lesser than all above-mentioned protocols during sparse network. In addition, EC is low when the network is dense. The reason is the fastest and shortest path selection (using the BF algorithm) and 3-hop neighbors checking.

It is clear from Figure 16 that both proactive protocols outperformed the counterpart as compared to benchmark routing protocols, i.e., Hydrocast, Co-Hydrocast and Co-Improved Hydrocast routing protocols.

#### 6.1.2. Impact of DA on EC

**Figure 17:**
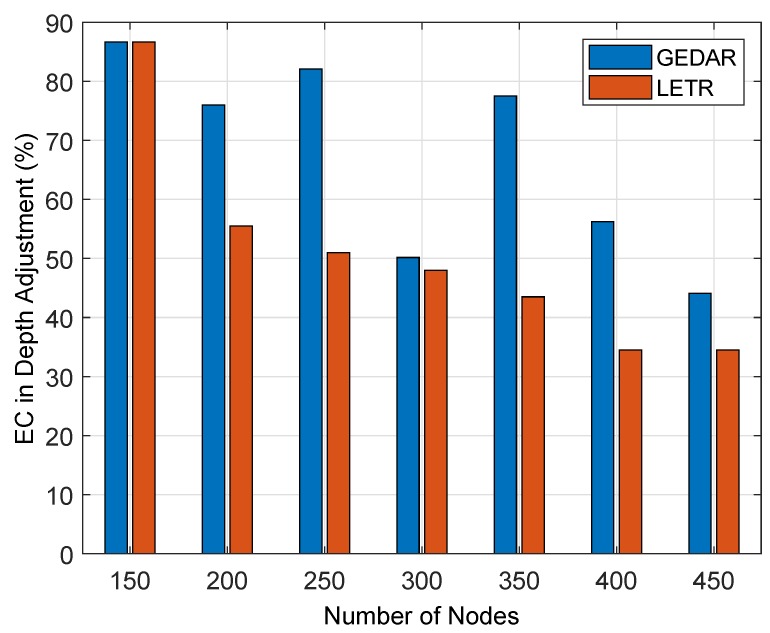
EC in DA.

Figure 17 illustrates EC during DA in benchmark protocols. During any change in the location of the sensor node, some percentage of energy is consumed. This EC decreases with the increase in nodes density. Two important things for the accountability of this EC are the network type (i.e., sparse or dense deployment) and nodes density (number of nodes). If the IoT-UWSN is spare, then the Probability of void hole increases. Decreasing behavior of EC is clearly depicted in Figure 17. The probability of void hole occurrence decreases with the increase in nodes density. This decrease in void hole results in few DAs, which lead the UWSN towards less EC. Energy consumed on DA in GEDAR and LETR is shown Figure 17. It is clear from the figure that during initial stages the EC of both aforementioned protocols was a bit high. Afterward, it reduces with increase in nodes density in IoT-UWSN.

#### 6.1.3. DA

**Figure 18:**
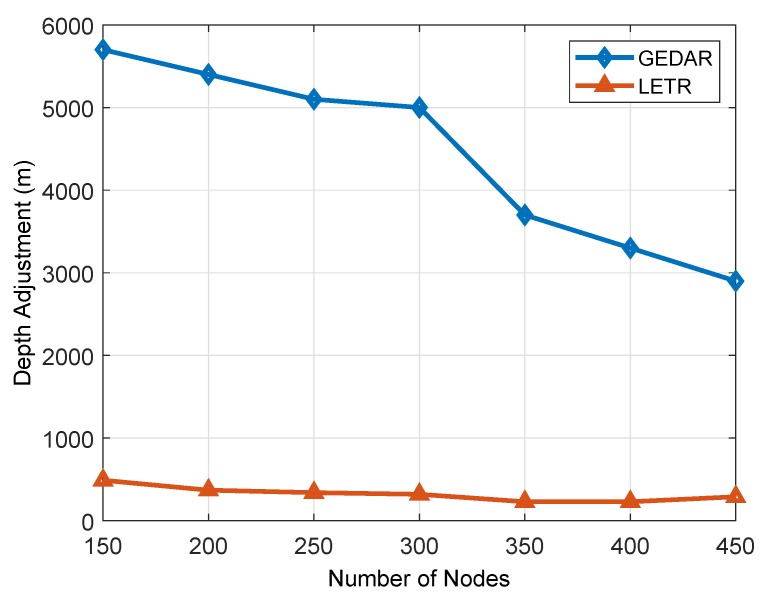
DA.

Figure 18 shows DA of both existing protocols, i.e., GEDAR and LETR. The reason to demonstrate DA of these two protocols is that this DA only happens in these two routing protocols. DA is the displacement of nodes from their real coordinates. This displacement mainly relies on nodes deployment. If the network is sparse, then the chances of void hole occurrence will be high and vice versa. GEDAR has higher DA than LETR because LETR has a minimum number of DAs because it also has the property of transmission range adjustment. Moreover, LETR prioritizes transmission range adjustment over DA. It is obvious from the figure that LETR performed fewer DAs than GEDAR.

#### 6.1.4. PDR

**Figure 19:**
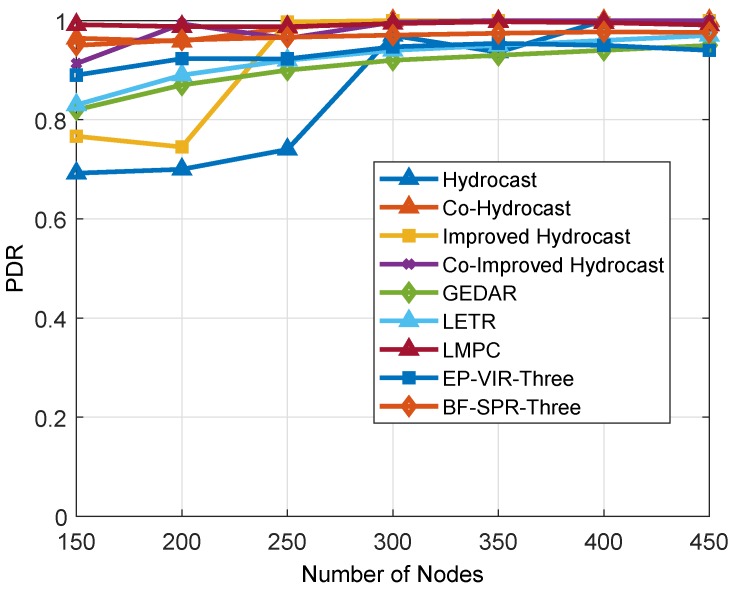
PDR.

PDR of the proposed and benchmark protocols is pectorally described in Figure 19. It is obvious from the figure that Hydrocast and Improved Hydrocast routing protocols have low PDR as compared to both benchmark and proposed protocols (during sparse IoT-UWSN). PDR of the protocols increases by increasing nodes density. The reason to enhance PDR in proactive protocols is that they minimize the void hole problem using shortest recovery path and opportunistic cooperation techniques (using fixed relay nodes).

While in the proposed protocols (EP-VIR-Three and BF-SPR-Three) the 3-hop neighbor’s information for the forwarder node selection helps the protocols in avoiding the void holes, this forwarder potential node selection helps the routing protocols to enhance their PDR. It can be clearly seen from the figure that Proposed protocols outperformed in PDR compared to benchmark protocols.

#### 6.1.5. Packet E2E Delay

**Figure 20:**
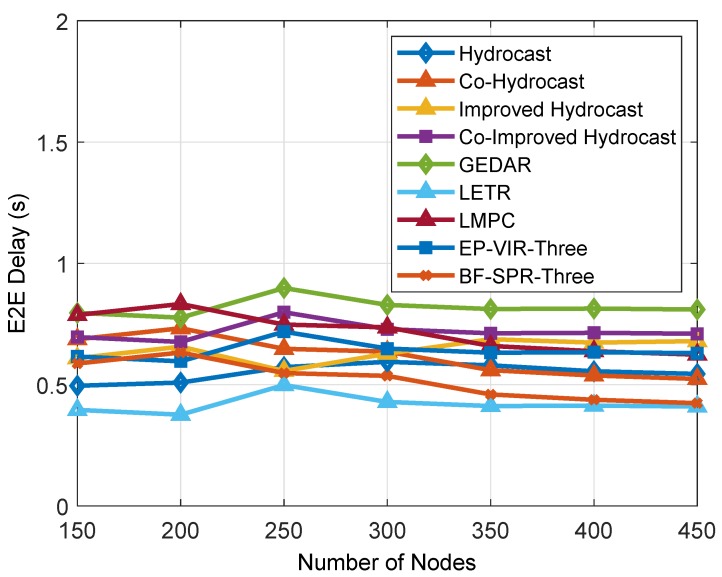
Packets E2E delay.

Packets E2E delay is shown in Figure 20. The GEDAR has high packet E2E delay due to its DA strategy. In addition, continuous DA in GEDAR results in high EC. This dissipation leads the network to originate a new void hole in that region. Then, GEDAR performs some more DAs to avoid the void hole in that region. This adjustment results in some more E2E delay. In addition, LMPC uses a layered multi path routing approach. This approach generates multiple copies of data and transmits over multiple paths using a binary tree approach. This strategy helps the network for minimum packet loss. However, LMPC protocol takes higher packet delay than LETR because of the collisions of multiple copies of DPs originating from cross nodes. However, this E2E delay is less than GEDAR and higher than LETR. The reason for less E2E delay of LETR is that LETR prioritizes the transmission range adjustment over DA and has no multiple copies of data.

Meanwhile, both protocols well performed in minimizing the packets E2E delay than benchmark proactive routing protocols (i.e., Hydrocast, Co-Hydrocast, Improved Hydrocast and Co-Improved hydrocast). EP-VIR-Three provides an efficient routing path with no collision and interference (using 3-hop neighbor’s information and CF value). This routing path includes the forwarders node having a minimum number of neighbors with minimum hop counts from the source node to the sink node, while, in BF-SPR-Three, the packets E2E delay is less than LMPC, GEDAR, EP-VIR-Three, Improved Hydrocast and Co-Improved Hydrocast routing protocols because of shortest and efficient route selection using 3-hop neighbor’s information and binary tree generation (the shortest path is originated through Bellman–Ford algorithm).

### 6.2. Performance Trade-Off

In this subsection, the trade-off between the benchmark and the proposed protocols is discussed. In benchmark protocols, LETR performs error resilient transmission range adjustment that consumes high EC by compromising E2E delay. Therefore, the protocol has compromised its E2E delay by avoiding the void hole problem to provide error resilient transmission. While in Hydrocast routing protocol [26], PDR is achieved on the cost of E2E delay. Moreover, Improved Hydrocast protocol has compromised its EC on fixed nodes deployment by paying the cost E2E delay. In addition, Co-Hydrocast routing protocol improved its PDR by paying the cost on opportunistic and cooperative routing. In the end, Co-Improved Hydrocast routing uses both features including opportunistic and cooperative routing and fixed nodes deployment to avoid the void hole. Therefore, this protocol has compromised its EC on the cost of E2E delay. While in GEDAR, void hole avoidance is tackled on the base of DA of the nodes which consumes high EC, there exists a trade-off between EC and E2E delay. In addition, LMPC uses a tree based multi path routing approach for reliable data delivery. In this protocol, EC and the active number of nodes are compromised over packet received ratio.

In the proposed protocol EP-VIR-Three, a proactive routing approach is implemented to search the next forwarder node using 3-hop neighbor’s information. Therefore, EP-VIR-Three has compromised reliable data delivery by paying affordable E2E delay, resulting in high PDR. Meanwhile, BF-SPR-Three selects the single shortest and fastest path for reliable data delivery (additionally the binary tree generation) on the cost of affordable E2E delay. Moreover, performance trade-off and achievements with compromised parameters of benchmark and the proposed protocols are shown in Table 5.

## 7. Conclusions

In this work, void hole problem is alleviated using two proactive routing protocols. These routing protocols perform reliable and interference-free routing (using the greedy approach) in IoT-UWSN. Both protocols use sender based approach to minimize the EC and E2E delay. The proposed protocol EP-VIR-Three selects the next forwarder node with a minimum number of neighbors (to provide collision and interference-free communication using greedy forwarding) using 3-hop neighbors information. Meanwhile, the proposed protocol BF-SPR-Three selects the fastest and shortest path using the ‘BF’ algorithm (to provide reliable data delivery with the binary tree generation approach for greedy forwarding) using 3-hop neighbors information. Additionally, the feasible regions using linear programming are also computed for optimal EC and to improve the network lifetime. Moreover, the data forwarding algorithms for the proposed routing protocols are also presented. The scalability of the proposed routing protocols is also analyzed by varying the number of nodes. In the end, comparative analysis is performed with benchmark routing protocols. It is evident that the EC of the proposed protocols is minimized (25–56% of the state-of-the-art routing protocols) in a dense network. And, outperformed the benchmark routing protocols in counterparts.

### Future Work

In the future, the proposed protocols will be exploited further to implement some artificial intelligence techniques to avoid the void hole in IoT-UWSN. Implementing these techniques on the testbed will be our new research direction for getting more precision in data reliability. In addition, the proposed routing protocols do not reflect quickly during the dynamic topological changes in the IoT-UWSN. Therefore, the handling of these issues will also be part of our future directions. Furthermore, the density of the simulation is too high in our research work, which will be further optimized in our future work.

## Figures and Tables

**Table 1:** Summarized and Categorized Literature Review.

Categorization	Protocols	Achievements	Limitations
Energy based	RE-PBR [8]	An energy efficient multi-hop protocol with maximum PDR and data reliability	Dense Deployment of the network is challenge with high E2E delay
	TCEB [9]	A multi-hop protocol with minimum EC	Increase in E2E delay and EC due to attenuation is not focused
	EBLE [10]	A single-hop protocol to balance the traffic load for minimum EC with reliable data delivery and packet size management	EC due to delay and path loss due to continuous packet transmission is not focused
	Cooperative routing protocol [11]	A single-hop protocol with minimum EC and high PDR	Sparse region effect is ignored by the authors
	EBULC [19]	A multi-hop routing for minimum EC	Network complexity is a challenge with affordable E2E delay
	Review of state-of-the-art protocols [21]	Single and multi-hop routing protocols with reliable data delivery and mobility management approach	Security issues and high EC
Topology based	TCEB [9]	A multi-hop routing with minimum EC and adaptive for dynamic topology	E2E delay and communication overhead is not focused
	Classifying topology control based algorithm [12]	Energy efficient topology	Mobility management is not focus by the authors
	GARM [13]	A single hop routing with minimum EC with enhanced PDR	Proposed protocol only performed efficient in predefined environments
Void Node based	TORA [22]	A multi-hop routing with minimum ed to end delay and void hole avoidance	Computational time is not focused
	GEDAR [23]	Void hole is successfully removed	EC for the DA is increased
	LMPC [24]	Void hole alleviation	Communication overhead due to multiple copies is discussed

**Table 2:** Format of Hello packet.

Node ID	Number of Neighbour Nodes	Distance from Sink	Number of Hops from Sink

**Table 3:** Format of routing table.

Neighbor ID	Total Number of Neighbors in its Transmission Range	Distance from Neighbors	Number of Hop Counts from Sink

**Table 4:** Simulation parameters.

Parameters	Values
Number of Sensor Nodes	150 to 450
Number of Sinks	9
Initial Energy of the Nodes (J)	100
Network Area (m${}^{3}$)	1500 × 1500 × 1500
Transmission Range of Nodes (m)	250
Transmission Power (W)	2
Reception power (W)	0.1
Idle State Power (mW)	10
Data Size (Kbps)	50
Packet Size (bytes)	200
Noise of Ship (db)	0.2
Bandwidth (KHz)	4
Beacon (bites)	50
Wind (m/s)	5
Node Speed in the Horizontal Direction (m/s)	2
Layers	6
Frequency	914 × $10^{6}$ Hz
Energy for DA (mJ/m)	1500
Mobility Model	Random Walk 2D Mobility Model

**Table 5:** Performance trade-offs between benchmark and the proposed protocols.

Protocols	Achievements	Compromised Parameters
LETR [14]	Void hole avoidance and error resilient communication	Network faces high EC and high E2E delay
Hydrocast [26]	PDR is achieved	Network has faced E2E delay
Improved Hydrocast [27]	Network faces less E2E delay	EC on fixed nodes deployment
Co-Hydrocast [27]	Network faces high PDR	Network faces high EC due to opportunistic cooperative routing
Co-Improved Hydrocast [27]	Network faces low E2E delay with high PDR	EC on fixed nodes deployment and on opportunistic cooperative routing
GEDAR [23]	Void hole avoidance	High EC during void hole avoidance with affordable E2E delay
LMPC [24]	Reliable DPs transmission	High EC due to binary tree generation from the source node
EP-VIR-Three	Void hole avoidance with reliable data delivery	EC during interference and collision-free path selection with affordable E2E delay
BF-SPR-Three	Void hole avoidance and reliable data transmission (using shortest and fast path given by bellman ford algorithm)	EC due to binary tree generation with affordable E2E delay

## Abbreviations

The following abbreviations are used in this manuscript:

IoT-UWSN	Internet of Things enabled Underwater Wireless Sensor Network
UWSN	Underwater Wireless Sensor Network
EC	Energy Consumption
E2E	End to End
BF	Bellman–Ford
BF-SPR-Three	Bellman–Ford Shortest Path-based Routing
EP-VIR-Three	Energy-efficient Path-based Void hole and Interference-free Routing
GEDAR	GEographic and opportunistic routing with Depth Adjustment-based topology control for communication Recovery
LETR	Location Error-resilient Transmission Range adjustment-based protocol
LMPC	Layered Multi path Power Control
DBR	Depth Base Routing
DA	Depth Adjustment
WDFAD-DBR	Weighting Depth Forwarding Area Division DBR
NR	Noise Resources
DP	Data Packet
cc	Channel Capacity
Freq	Frequency
PL	Packet Length
NP	Total Noise Power
SNR	Signal-to-Noise Ratio
CF	Cost Function
THCs	Total Hop Counts
**Acronyms**	
Dis	Distance from n−1 to *n*th node
*B*	Bandwidth of the acoustic channel
$H_{2}$	Binary entropy function using PER
*P*	The power of the acoustic signal
$T_{p}$	Transmission power of the sensor node
$R_{p}$	Reception power of the sensor node
PS	Packet size
DR	Data rate of the acoustic channel
DP	Data packets
$R_{t}$	Transmission range
$E_{i}$	The initial energy of the node
$T_{E}$	Total energy of the network
*N*	Total number of DPs in the network
$H_{2}$	Binary entropy function
*P*	Path
tn	Total noises
P(t)	Packet type
$E_{trans}$	EC of the sensor nodes during the packet transmission
$E_{rec}$	EC of the sensor nodes during the packet reception
PER	The number of bits without any error
$t_{tot}$	Total time of transmission
$E_{consumption}$	Total energy consumed
$E_{rec}^{rem}$	Total residual energy of the nodes after receiving all the DPs
$E_{trans}^{rem}$	Total residual energy of the nodes after forwarding all the DPs
